# Combined Therapy with Simvastatin- and Coenzyme-Q10-Loaded Nanoparticles Upregulates the Akt-eNOS Pathway in Experimental Metabolic Syndrome

**DOI:** 10.3390/ijms24010276

**Published:** 2022-12-23

**Authors:** Ezgi Şaman, Martina Cebova, Andrej Barta, Martina Koneracka, Vlasta Zavisova, Anita Eckstein-Andicsova, Martin Danko, Jaroslav Mosnacek, Olga Pechanova

**Affiliations:** 1 Institute of Normal and Pathological Physiology, Centre of Experimental Medicine, Slovak Academy of Sciences, 813 71 Bratislava, Slovakia; 2 Institute of Experimental Physics, Slovak Academy of Sciences, 040 01 Kosice, Slovakia; 3 Polymer Institute, Slovak Academy of Sciences, 845 41 Bratislava, Slovakia; 4 Centre for Advanced Materials Application, 845 11 Bratislava, Slovakia; 5 Institute of Pathophysiology, Faculty of Medicine, Comenius University, 811 08 Bratislava, Slovakia

**Keywords:** statins, polymeric nanoparticles, nitric oxide synthase, Akt-eNOS pathway, nicotinamide adenine dinucleotide phosphate oxidase, nuclear factor kappaB, cardiometabolic diseases

## Abstract

In addition to their LDL-cholesterol-lowering effect, statins have pleiotropic beneficial effects on the cardiovascular system. However, long-term treatment with statins may be associated with serious side effects. With the aim to make statin therapy more effective, we studied the effects of simvastatin- and coenzyme-Q10-loaded polymeric nanoparticles on the lipid profile and nitric oxide (NO)/reactive oxygen species (ROS) balance in the heart and aorta of adult male obese Zucker rats. The rats were divided into an untreated group, a group treated with empty nanoparticles, and groups treated with simvastatin-, coenzyme Q10 (CoQ10)-, or a combination of simvastatin- and CoQ10-loaded nanoparticles (SIMV+CoQ10). After 6 weeks, the lipid profile in the plasma and the concentration of conjugated dienes in the liver were determined. Nitric oxide synthase (NOS) activity, Akt, endothelial NOS (eNOS), phosphorylated eNOS (p-eNOS), nicotinamide adenine dinucleotide phosphate (NADPH) oxidase, and nuclear factor kappaB (NF-kappaB) protein expressions were measured in the heart and aorta. All simvastatin, CoQ10, and SIMV+CoQ10 treatments decreased plasma LDL levels, but only the combined SIMV+CoQ10 treatment increased NOS activity and the expression of Akt, eNOS, and p-eNOS in both the heart and the aorta. Interestingly, NADPH oxidase in the heart and NF-kappaB protein expression in the aorta were decreased by all treatments, including nanoparticles alone. In conclusion, only combined therapy with SIMV- and CoQ10-loaded nanoparticles increased NOS activity and upregulated the Akt-eNOS pathway in obese Zucker rats, which may represent a promising tool for the treatment of cardiometabolic diseases.

## 1. Introduction

Metabolic syndrome is a serious medical condition that increases the risk of heart disease, diabetes, stroke, and atherosclerosis. The underlying causes of metabolic syndrome include overweight, insulin resistance, physical inactivity, genetic factors, and increasing age. In general, metabolic syndrome comprises obesity, hypertension, high blood glucose, and dyslipidemia [1,2]. Elevated production of free radicals leading to oxidative stress and impaired nitric oxide (NO)/reactive oxygen species (ROS) balance is the earliest step in the development of the pathophysiology of metabolic syndrome [3,4,5]. Mechanisms of impaired NO bioavailability include mainly increased production of ROS, resulting in the formation of peroxynitrite, endothelial nitric oxide synthase (eNOS) uncoupling, and diminished regulation of eNOS phosphorylation, such as phosphatidylinositol-3-kinase-Akt (PI3K-Akt) signaling [6,7]. Akt, together with 5’ adenosine monophosphate-activated protein kinase (AMPK), extracellular signal-regulated protein kinase (ERK1/2), and Ca2+/calmodulin-dependent protein kinase II (CaMK-II), phosphorylates eNOS at Ser1177 leading to increased eNOS activity [6,8,9,10]. A deficiency in Akt/eNOS signaling may indicate impaired insulin signaling in type 2 diabetes, as in the experimental diabetic animals and in type 2 diabetic patients Akt activity and eNOS phosphorylation were decreased [11,12]. Reduced eNOS Ser1177 phosphorylation associated with vascular dysfunction and elevated blood pressure has been demonstrated in high-fat-fed mice [13] and SHRSP.Z-Leprfa/IzmDmcr rats with metabolic syndrome [14]. These results clearly demonstrate that the Akt-eNOS pathway plays a serious role in different symptoms of metabolic syndrome. Statins are among the drugs of first choice used to treat the metabolic derangements produced by metabolic syndrome. Synthetic and natural statins have essentially equivalent efficacy at improving the lipid profile. However, in patients who achieve decreased low-density lipoprotein (LDL) levels with difficulty, atorvastatin and simvastatin may be the best choices [15]. Numerous studies have demonstrated that statins enhance NOS activity and NO production by affecting different signaling pathways [16]. Decreasing LDL cholesterol and caveolin-1, a negative regulator of eNOS, represent the cholesterol-dependent effect of statins on NO generation [17]. Cholesterol-independent effects include eNOS upregulation [18,19,20], increased eNOS phosphorylation at Ser 1177 [21,22,23], and inhibition of nicotinamide adenine dinucleotide phosphate (NADPH) oxidase and nuclear factor kappaB (NF-kappaB) resulting in decreased oxidant and proinflammatory status [24,25]. Recent research does not clearly suggest that one statin is better than another at causing these effects [15].

However, long-term statin use may be associated with considerable residual risk and several side effects [26]. Reducing endogenous CoQ10 production is one of the most significant side effects of statins. CoQ10 is an essential compound in the human body, and its deficiency causes disruption of cellular energy metabolism and contributes to the development of myopathy and other muscle disturbances. On the other hand, under metabolic syndrome conditions, CoQ10 supplementation decreases ROS production and improves glucose metabolism, triglycerides, total cholesterol, and LDL levels [27,28]. 

Moreover, negative properties of statins include poor water solubility, low bioavailability, and relatively high doses of treatment. A novel approach to improve the bioavailability and stability of statins is using the nano-encapsulated form or drug-loading nanoparticles. A proper nano-encapsulation can improve the drug’s solubility while decreasing its side effects leading to an overall improvement in the therapeutic efficacy [26,29,30,31]. It has been documented that statin-loaded polymeric nanoparticles displayed a superior profile concerning the bioavailability, drug release, dosing, and minimizing adverse effects [26,32]. In a hyperlipidemic rat model, administration of atorvastatin-loaded polymeric nanoparticles every 3 days exhibited the same efficacy as the once-daily treatment with a commercial formulation of atorvastatin. As a result, the daily dose of atorvastatin was reduced by 66% using polymeric nanoparticles [32]. In this context, polymeric nanoparticles may represent a promising tool for statin nano-encapsulation and efficient treatment [26,33]. Bioavailability varies widely between statins. Simvastatin has less than 5 percent bioavailability but is recommended for patients who have more difficulty in lowering LDL [15].

To make simvastatin therapy more effective, we studied the effects of combined treatment with simvastatin- and CoQ10-loaded polymeric nanoparticles on lipid profile and NO/ROS balance in the heart and aorta of adult male obese Zucker rats. To our best knowledge, this is the first study to investigate the combined therapy with simvastatin- and CoQ10-loaded polymeric nanoparticles using a copolymer of poly(ethylene glycol) methacrylate (PEGMA) with N-vinyl-2-pyrrolidone (VP) and N-octadecyl methacrylamide (OMA). While the use of CoQ10 may reduce the side effects of simvastatin associated with decreased synthesis of this substance during simvastatin therapy, nano-encapsulation may prolong the bioavailability of simvastatin leading to the overall improvement of the therapeutic efficacy.

## 2. Results

### 2.1. Nanoparticle Characteristics 

**Figure 1:**
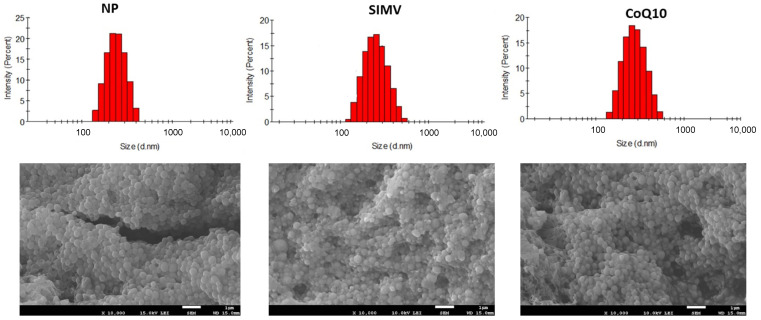
Graphic presentation of particle size according to the intensity obtained from dynamic light scattering (DLS) and pictures from scanning electron microscopy (SEM) for empty nanoparticles (NP) and NP loaded with simvastatin (SIMV) or coenzyme Q10 (CoQ10).

Random co-polymerization of PEGMA with VP and OMA was performed using the free radical polymerization process of micellar aggregates formed from the monomers [34]. The final NP exhibited an amphiphilic character, due to the character of the monomers used. The size and size distribution (PDI) of the pure and loaded NP were determined with dynamic light scattering (DLS) in an aqueous dispersion at 25 °C and the results are summarized in the Table 1. Under the described experimental conditions the NP with a size of 233 nm was obtained. After loading with simvastatin and CoQ10 the size increased to 347 nm and 270 nm, respectively. The size distribution of pure NP was narrow and became a little bit broader after loading. The zeta potentials determined for all NP showed a partial negative charge at the NP surface with values showing moderate dispersion stability, which was increased after loading of NP with SIMV. Analysis of the morphology of the prepared NP before and after loading with the bioactive compounds showed a spherical-shaped formation of nanoparticles (Figure 1).

Using the dialysis bag method it was found that 180 mg of simvastatin and 200 mg of coenzyme Q10 were released from 1 g of NP after 48 h. Such a quantity was calculated when designing in vivo experiments.

### 2.2. Weight Parameters 

There were no significant changes in BW, HW, LKW, relative HW, and relative LKW between the groups (Table 2). 

### 2.3. Lipid Profile and Lipid Peroxidation

Simvastatin-loaded nanoparticles decreased total cholesterol, HDL, and LDL while combined therapy- and CoQ10-loaded nanoparticles decreased LDL and the concentration of hepatic conjugated dienes, compared to the respective parameters in the control obese Zucker rats. Interestingly, empty nanoparticles increased triglycerides (TG) and decreased total cholesterol and HDL levels compared to the control parameters (Table 3).

### 2.4. Protein Expressions of Akt, eNOS, and p-eNOS

**Figure 2:**
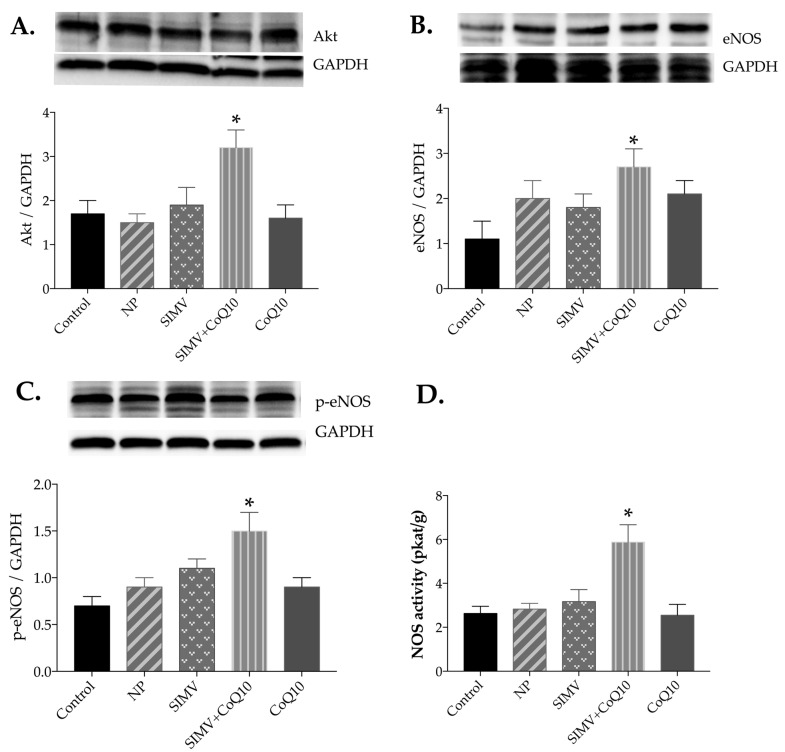
Expression levels and representative Western blot images of Akt (**A**), endothelial nitric oxide synthase (eNOS) (**B**), phosphorylated eNOS (p-eNOS) (**C**), and total NOS activity (**D**) in the heart of the control group and groups treated with empty nanoparticles (NP), simvastatin-loaded nanoparticles (SIMV), combination of simvastatin-loaded and coenzyme-Q10-loaded nanoparticles (SIMV+CoQ10), and coenzyme-Q10-loaded nanoparticles (CoQ10). * *p* < 0.05 compared to the control obese Zucker rats. Data are means ± SEM from 6 animals in each group.

**Figure 3:**
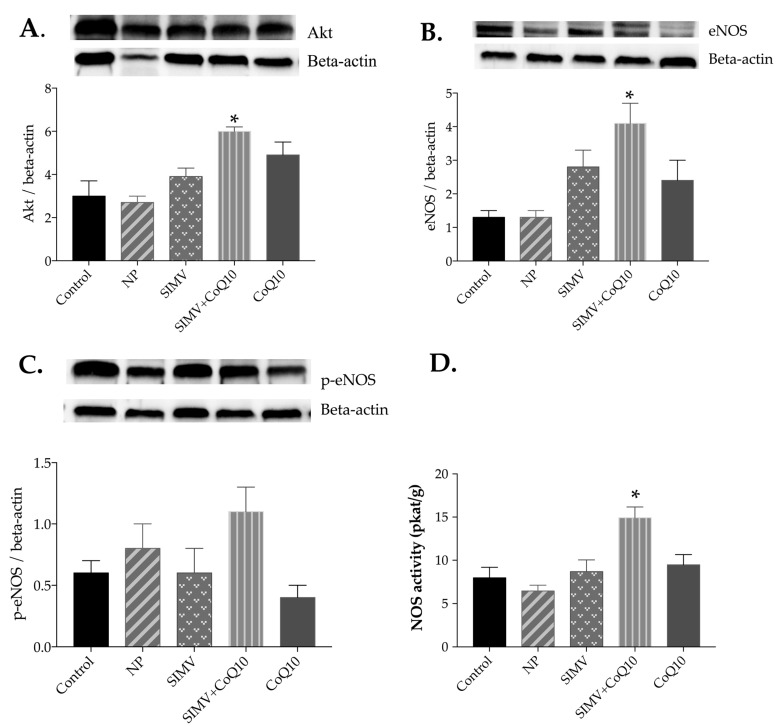
Expression levels and representative Western blot images of Akt (**A**), endothelial nitric oxide synthase (eNOS) (**B**), phosphorylated eNOS (p-eNOS) (**C**), and total NOS activity (**D**) in the aorta of the control group and groups treated with empty nanoparticles (NP), simvastatin-loaded nanoparticles (SIMV), combination of simvastatin-loaded and coenzyme-Q10-loaded nanoparticles (SIMV+CoQ10), and coenzyme-Q10-loaded nanoparticles (CoQ10). * *p* < 0.01 compared to the control obese Zucker rats. Data are means ± SEM from 6 animals in each group.

Only the combined therapy with simvastatin- and CoQ10-loaded nanoparticles was able to increase protein expression of Akt, eNOS, and p-eNOS in the heart tissue (Figure 2A–C). Similarly, combined therapy increased protein expression of Akt and eNOS in the aorta compared to the respective parameters in the control obese Zucker rats (Figure 3A–C).

### 2.5. Total NOS Activity

Only the combined therapy with simvastatin- and CoQ10-loaded nanoparticles was able to increase NOS activity in the heart and aorta compared to control NOS activity in the obese Zucker rats (Figure 2D and Figure 3D).

### 2.6. Protein Expressions of NADPH Oxidase and NF-kappaB

**Figure 4:**
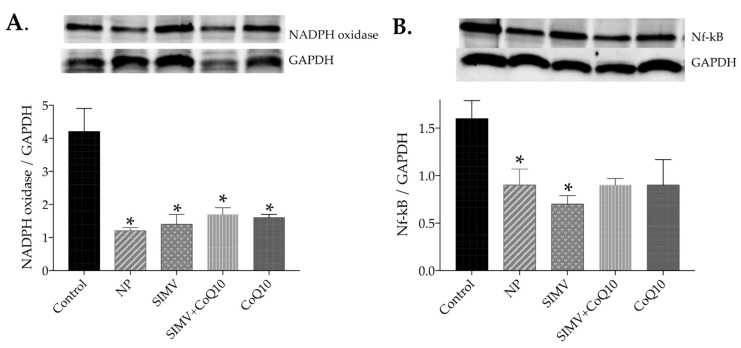
Expression levels and representative Western blot images of nicotinamide adenine dinucleotide phosphate (NADPH) oxidase (**A**) and nuclear factor kappaB (NF-kappaB) (**B**) in the heart of the control group and groups treated with empty nanoparticles (NP), simvastatin-loaded nanoparticles (SIMV), combination of simvastatin-loaded and coenzyme-Q10-loaded nanoparticles (SIMV+CoQ10), and coenzyme-Q10-loaded nanoparticles (CoQ10). * *p* < 0.001 compared to the control obese Zucker rats. Data are means ± SEM from 6 animals in each group.

**Figure 5:**
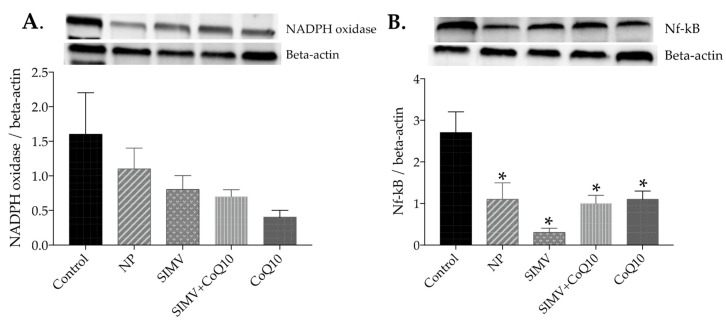
Expression levels and representative Western blot images of nicotinamide adenine dinucleotide phosphate (NADPH) oxidase (**A**) and nuclear factor kappaB (NF-kappaB) (**B**) in the aorta of the control group and groups treated with empty nanoparticles (NP), simvastatin-loaded nanoparticles (SIMV), combination of simvastatin-loaded and coenzyme-Q10-loaded nanoparticles (SIMV+CoQ10), and coenzyme-Q10-loaded nanoparticles (CoQ10). * *p* < 0.01 compared to the control obese Zucker rats. Data are means ± SEM from 6 animals in each group.

All treatments decreased NAPDH oxidase protein expression in the heart (Figure 4A) while none of the treatments had an effect in the aorta (Figure 5A). Simvastatin-loaded nanoparticles and empty nanoparticles decreased NF-kappaB protein expression in the heart (Figure 4B) and all treatments decreased it in the aorta compared to control obese Zucker rats (Figure 5B).

## 3. Discussion

According to the literature, simvastatin is a long-known hydroxy-methylglutaryl coenzyme A reductase inhibitor with a maximum recommended dose of 80 mg/day. It causes an average reduction in low-density lipoprotein cholesterol (of about 45%), accompanied by a decrease in very low-density lipoprotein cholesterol, triglycerides, and apolipoprotein B [35,36]. The most serious side effect of simvastatin is myopathy; when severe, it can take the form of rhabdomyolysis, often leading to acute renal failure [37]. The reduction in CoQ10 that accompanies simvastatin administration has been shown to be at least partly responsible for this side effect [38]. On the other hand, recent studies have provided evidence of positive lipid-independent pleiotropic effects of simvastatin. These pleiotropic effects involve the improvement of cardiovascular and endothelial functions, enhancing the stability of atherosclerotic plaques, and beneficial effects on the immune system, central nervous system, and bones [39]. Some of these effects may be mediated by the inhibition of isoprenoids—the important intermediates of lipid attachments for posttranslational modifications of different signaling proteins, such small GTP-binding proteins, Rho, Ras, and Rac [39]. Many pleiotropic effects, particularly within the cardiovascular system, include Akt activation, eNOS upregulation, and Ser 1177 phosphorylation [17,20,21,22]. Also, the antioxidant and anti-inflammatory properties of simvastatin are documented in different tissues [24].

Attenuation of the negative and strengthening of the positive effects of drugs can be achieved by more effectively targeted therapy through nano-encapsulation or binding of the drug to polymeric nanoparticles. [33,40,41,42]. To our best knowledge, we have shown for the first time that only combined therapy using both the simvastatin-loaded and CoQ10-loaded polymeric nanoparticles upregulated the Akt-eNOS pathway in the heart and aorta of obese Zucker rats. Recently, we have revealed an increase in aortic eNOS protein expression after pure simvastatin treatment in hereditary hypertriglyceridemic rats [43]. Similarly, in N^G^-Nitro-L-arginine methyl ester (L-NAME)-induced hypertension simvastatin improved NO production and partially prevented hypertension development [44]. The data of Kureishi et al., 2000 also showed that simvastatin and pravastatin rapidly induced phosphorylation of Akt at serine residue 473 which increased its protein kinase activity. Furthermore, simvastatin-induced Akt-mediated phosphorylation of eNOS leads to NO production [23]. Simvastatin, however, did not increase NO production already enhanced by other interventions [19,45]. Fluvastatin treatment in the cholesterol-fed rabbits increased eNOS mRNA expression and decreased superoxide production in the endothelial cells of the aorta accompanied by an increase in cyclic GMP concentration [46]. Similarly, eNOS activity was enhanced markedly in mice treated with cerivastatin, and the angiogenic effect of cerivastatin was dismissed in eNOS-deficient mice [47]. Both pitavastatin and rosuvastatin upregulated eNOS and increased NO production via activating eNOS phosphorylation at Ser-1177 in human umbilical vein endothelial cells [48,49]. Rosuvastatin also reversed diminished NO-dependent vasorelaxation responses in the aorta in streptozotocin-induced diabetic mice without affecting total plasma cholesterol levels [50]. These data obtained from pure statin treatments are consistent with our findings since according to our results statin-loaded nanoparticle therapy may have a similar effect but requires the addition of CoQ10. In our experiment, the ratio of p-eNOS/eNOS in the control group and SIMV+CoQ10 group was similar. However, the treatment increased the expression not only of eNOS but also of p-eNOS, i.e., the active part of eNOS. This means that the SIMV+CoQ10 treatment had a significant effect at the translational level (increased eNOS protein expression) and posttranslational level (increased eNOS phosphorylation). The ratio indicates only the activation of the protein; however, an increase in phosphorylation means an increased activity of eNOS. Even if the protein has the same activation, its absolute amount has increased, therefore the activity is ultimately increased. This fact was also confirmed by the increased activity of NOS, which was measured by the formation of [3H]-L-citrulline from [3H]-L-arginine in the heart and aorta.

CoQ10 plays a key role in the electron transport chain by providing an efficient supply of energy. Moreover, CoQ10 has strong antioxidant and anti-inflammatory properties, thus preventing free-radical-induced damage and inflammatory signaling pathway activation [51,52]. Indeed, in our experimental conditions, treatment with CoQ10-loaded nanoparticles decreased the expression of NADPH oxidase in the heart and NF-kappaB in the aorta. Moreover, CoQ10-loaded nanoparticles decreased LDL as well as the concentration of hepatic conjugated dienes, thus confirming the inhibitory effect on lipid peroxidation. The antioxidant effect of simvastatin-loaded nanoparticles has also been demonstrated in our experimental conditions. Simvastatin-loaded nanoparticles decreased the protein expression of NADPH oxidase in the heart and NF-kappaB protein expression in both heart and aorta. Similarly, statin enhanced endothelium-dependent relaxation by inhibiting the production of ROS and reduced vascular superoxide generation in cholesterol-fed rabbits [53]. Simvastatin reduced intracellular ROS levels, inhibited the activation of the NADPH oxidase/p38 mitogen-activated protein kinase (MAPK) pathway, and decreased NF-kappaB nuclear transcription in a mouse Parkinson’s disease model [24]. Despite these facts, in our experimental conditions only combined therapy with simvastatin- and CoQ10-loaded nanoparticles upregulated the Akt-eNOS pathway. We hypothesized that enhancing the pleiotropic effects of simvastatin with the antioxidant properties of CoQ10 may increase the activating effect on the Akt-eNOS pathway and improve NO/ROS balance. Decreased levels of ROS may lead to the stabilization of the eNOS dimer and better-phosphorylated conditions [54].

The exploitation of the pleiotropic effects of statins has been greatly hindered by poor bioavailability and adverse effects on muscles and the liver at higher doses [55,56,57]. Therefore, we have prepared simvastatin-loaded polymeric nanoparticles and studied their effects together with CoQ10-loaded nanoparticles. In our experimental conditions 180 mg of simvastatin and 200 mg of coenzyme Q10 were released from 1 g of polymeric nanoparticle after 48 h. Such a quantity was calculated when designing in vivo experiments. The polymeric nanoparticles were stable at gastric and oral pH.

Our results regarding the in vivo treatment with empty polymeric nanoparticles also provided notable findings. Empty polymeric nanoparticles decreased total cholesterol and HDL levels compared to controls. In relation to HDL, loading nanoparticles with CoQ10 abolished this effect. Some studies have shown increased HDL levels after CoQ10 treatment [58]. This may probably be the reason why CoQ10-loaded nanoparticles did not reduce HDL levels in our experiment. Similarly, like simvastatin-loaded nanoparticles, empty nanoparticles decreased the expression of NADPH oxidase in the heart and NF-kappaB protein expression in the heart and aorta. The shell part of the nanoparticles consists mainly of poly(ethylene glycol) (PEG) chains with a small content of pyrrolidone groups. Both PEG and poly(N-vinyl pyrrolidone) are commonly used in various biomedical studies and different PEG-dependent beneficial effects have already been described. PEG itself may interact with biological membranes and has a membrane-protective effect [59,60]. Recently it has been reported that PEG interacted with glycerophospholipids in monolayers and diminished oxidative stress, thus maintaining cell membrane integrity [61]. Ferrero-Andres demonstrated the anti-inflammatory effect of PEG in an experimental model of acute necrotizing pancreatitis. PEG proved to be particularly effective against associated acute lung inflammation [59]. The same study group showed the direct anti-inflammatory effect of PEG on macrophage activation. PEG significantly decreased interleukin-1-beta mRNA and protein expression. The authors hypothesized that the anti-inflammatory effect of PEG might be related to an interaction with the NF-kappaB signaling pathway [62].

Also, our results may imply that neither simvastatin nor CoQ10, but polymeric nanoparticles may be responsible for reducing the expression of NADPH oxidase and NF-kappaB. However, empty nanoparticles have a different size (233 nm) than simvastatin- (347 nm) or CoQ10-loaded nanoparticles (270 nm). Loading the nanoparticle with a drug always changes its size, making it difficult to compare the groups. Nevertheless, the size of simvastatin- or CoQ10-loaded nanoparticles and empty nanoparticles was as similar as possible.

However, the different size of the nanoparticles may be one of the limitations of our study. The second could be the fact that the activity of NOS measured by the formation of [3H]-L-citrulline from [3H]-L-arginine indicates the total activity and also includes neuronal (nNOS) and inducible nitric oxide synthase (iNOS) activities. Regarding iNOS, however, we do not expect its increase, since the inflammatory factor NF-kappaB was rather reduced after the SIMV+CoQ10 treatment. The third limitation may be the non-use of a group with pure simvastatin in our study; however, simvastatin is lipophilic, and its dissolution would introduce additional complications and increase the groups in the experiment.

In several studies, it has also been shown that statin nano-therapy using different nanotechnology systems may reduce or eliminate common adverse effects related to statin treatment [55,56,57]. Polymeric nanoparticles may help statin delivery and their positive pleiotropic effects by increasing oral bioavailability and enhancing target-specific interaction leading to reduced vascular endothelial dysfunction and increased cardiovascular regeneration [33]. Using polysialic acid-polycaprolactone, polymeric nanoparticle-based delivery of simvastatin was able to markedly reduce vascular smooth muscle cell chemotaxis and intimal hyperplasia [57]. Simvastatin-loaded poly-lactic-co-glycolic acid (PLGA) enhanced cell migration and growth factor expression in the experimental model of ischemic heart disease [63]. Pitavastatin-loaded poly-ethylene-glycol poly-lysin-phenylboronic acid nanoparticles even dose-dependently reduced aneurysm expansion [64]. Pitavastatin-loaded PLGA nanoparticles were able to repair injured vasculature via the activation of the PI3K signaling pathway promoting re-reendothelialization and reducing intimal hyperplasia [65]. Similarly, rosuvastatin-loaded poly(L-lactide-co-caprolactone) nanoparticles increased reendothelialization and reduced thrombotic potential via increased vascular endothelial growth factor signaling [66].

In conclusion, in our experimental study, only combined therapy using simvastatin-loaded nanoparticles together with CoQ10-loaded nanoparticles upregulated the Akt-eNOS pathway in obese Zucker rats. Enhancing the pleiotropic effects of simvastatin with the antioxidant properties of CoQ10 may increase the activating effect on the Akt-eNOS pathway and improve NO/ROS balance. Loading simvastatin and CoQ10 onto polymeric nanoparticles may improve their bioavailability and stability. For these reasons combined therapy with simvastatin- and coenzyme-Q10-loaded nanoparticles may represent a promising tool for the treatment of cardiometabolic diseases.

## 4. Materials and Methods

### 4.1. Chemicals

Most of the chemicals and reagents were obtained from Sigma-Aldrich (Saint-Louis, MO, USA); if not, the company is indicated. Simvastatin (99.7%) was isolated from the commercial drug Simvastatin-Ratiopharm 20 mg. CoQ10 was obtained from Tachyon Technology Pharm, Slovakia.

### 4.2. Simvastatin Extraction

The tablets of simvastatin were crushed, washed several times with water, and then extracted into dichloromethane at 40 °C. Subsequently, after evaporation and drying, pure simvastatin was obtained.

### 4.3. Synthesis and Loading of Polymeric Nanoparticles (NP)

**Figure 6:**
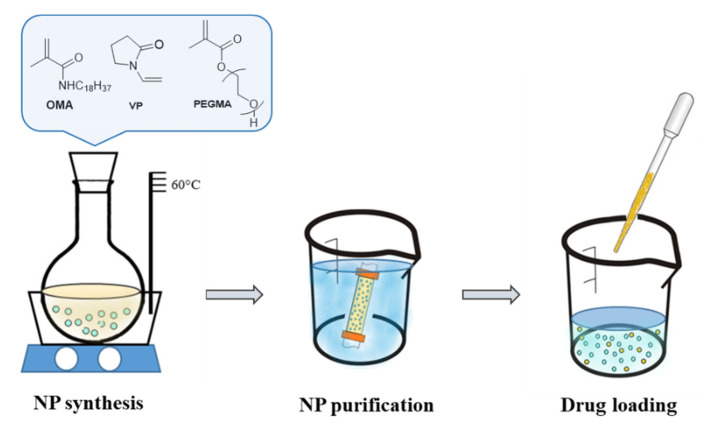
Schematic drawing of NP synthesis and loading.

The NP were synthesized and loaded according to Figure 6. A copolymer of poly(ethylene glycol) methacrylate (average Mn 360) with N-vinyl-2-pyrrolidone and N-octadecyl methacrylamide was synthesized through free radical polymerization. Before use, the PEGMA was released through the alumina column (Al2O3 basic) to remove the inhibitor and VP was freshly distilled. The OMA was synthesized according to the literature [67]. The PEGMA (4.1 mmol, 1.49 g) and VP (0.56 mmol, 60 µL) were added to the solution of OMA (2.9 mmol, 1 g) in isopropanol (33.3 mL). Water (66.7 mL) was added carefully to the reaction mixture and N, N-methylene bisacrylamide (MBA, 0.07 mmol, 0.011 g) as a crosslinker was added to the solution. The reaction mixture was purged with nitrogen gas for 30 min in order to remove the oxygen. Subsequently, ammonium persulfate (APS, 1.6 mmol, 0.365 g) and tetramethylethylenediamine (TEMED, 0.82 mmol, 123 µL) were added as the main initiation system, while ferrous ammonium sulphate (FAS, 0.6 mmol, 0.234 g) was added to activate the polymerization reaction. The polymerization mixture was stirred for 24 h at 60 °C. The reaction mixture was dialyzed (Spectrapore membrane 12–14 kDa) to the water overnight to remove any residual monomers. The dialyzed solution was then lyophilized to determine the weight of the prepared NP. As the reaction had to be repeated several times, on average 1.03 g of NP was obtained from each reaction.

The active component, i.e., simvastatin or CoQ10, was loaded onto the NP using the post-polymerization method as follows: The lyophilized NP were dispersed in 1 mL distilled water, while the active component was dissolved in acetone (4 wt %) and dropwise added to the constantly stirred NP dispersion. Subsequently, the loaded NP was lyophilized. The dried NP was carefully washed with acetone to remove the active components physically adsorbed on the NP surface and dried again. This prepared NP was used for in vivo experiments.

### 4.4. Physicochemical Characterization of the Simvastatin- and Coenzyme-Q10-Loaded Polymeric Nanoparticles

Particle size and zeta potential were determined using a Zetasizer Nano-ZS (Malvern Instruments, Malvern, UK) equipped with a helium/neon laser (λ = 633 nm) and thermoelectric temperature controller at a scattering angle of 173 deg and 25 °C. All the data analyses were performed in automatic mode. For the measurement, a concentration of 1 mg of NP in 1 mL distilled water was used. The morphology of the prepared NP before and after loading with the bioactive compound was monitored using scanning electron microscopy.

Drug encapsulation in amphiphilic polymer NP was conducted using the diffusion method. Several variables such as organic solvent, its ratio with the aqueous phase, drug to particle ratio, and mixing speed were optimized. Under the effect of process variables on mean particle size, drug loading was also screened. In vitro release of simvastatin and coenzyme Q10 from the loaded NP were investigated using the dialysis bag method. The release was studied in a solution of water:dioxane (1:3) after 12, 24, and 48 h of dialysis.

### 4.5. Animals and Treatment

All procedures and experimental protocols were approved by an Ethical Committee of the Centre of Experimental Medicine, Institute of Normal and Pathological Physiology, Slovak Academy of Sciences according to the European Convention for the Protection of Vertebrate Animals used for Experimental and other Scientific Purpose, Directive 2010/63/EU of the European Parliament.

Twelve-week-old male obese Zucker (fa-/fa-) rats were obtained from Charles River, USA. They were housed in groups of 2 animals under a 12 h light–12 h dark cycle at a constant humidity (45–65%) and temperature (20–22 °C). The rats were divided into the untreated group, the group treated with empty nanoparticles, and groups treated with or simvastatin- loaded NP, coenzyme-Q10-loaded NP, or a combination of SIMV-loaded NP and CoQ10-loaded NP. Each group consisted of 6 animals. All groups were fed with a standard diet ad libitum. Empty nanoparticles, simvastatin-loaded NP with a simvastatin dose of 15 mg/kg/day, coenzyme-Q10-loaded NP with a coenzyme Q10 dose of 15 mg/kg/day, or the combination were administered via the drinking water. Daily water consumption was estimated individually and adjusted, if necessary. The treatment lasted for 6 weeks.

### 4.6. Weight Parameters

At the end of the treatment, the animals were sacrificed; body weight (BW), heart weight (HW), tibia length (TL), and left kidney weight (LKW) were measured. Relative heart weight was calculated as an HW/TL ratio. Relative kidney weight was calculated as LKW/ 100 g BW.

### 4.7. Conjugated Dienes

Liver tissue was collected for the measurement of conjugated dienes. Samples of the liver were homogenized in 15 mmol/dm3 EDTA containing 4% NaCl. Lipids were extracted using a 1:1 chloroform–methanol mixture. Chloroform was evaporated in the N2 atmosphere and after the addition of cyclohexane, conjugated diene concentrations were determined spectrophotometrically (λ = 233 nm, NanoDrop 2000c, UV-Vis spectrophotometer, Thermo Fisher Scientific, Waltham, MA, USA). The concentration of CD was expressed as nmol per g of tissue.

### 4.8. Lipid Profile

Blood plasma was collected for measuring the lipid profile at the end of the treatment. The levels of triglyceride, total cholesterol, HDL, and LDL were measured with commercially available kits.

### 4.9. Western Blot Analysis

Tissue samples of the heart and aorta were homogenized, and the Western blot protocol was performed as previously described [68]. Membranes were incubated overnight with a primary polyclonal rabbit anti-pan-Akt (1:500, Abcam, ab8805), anti-eNOS (1:1000, Abcam, ab5589), anti-p-eNOS (1:1000, Invitrogen, #PA5-35879), anti-NADPH oxidase 4 (1:2000, Abcam, ab154244), and anti-NF-kappaB p65 (1:1000, Abcam, ab16502) antibodies as well as anti-GAPDH (1:5000, Abcam, ab201822) and anti-β-actin (1:2000, Abcam, ab8227) as a loading control. Antibodies were detected using a secondary peroxidase-conjugated goat anti-rabbit antibody (1:5000, Abcam, ab97051) at room temperature for 2 h. The intensity of bands was visualized using the enhanced chemiluminescence system (ECL, Amersham, UK), quantified using a ChemiDocTM Touch Imagine System (Image LabTM Touch software, BioRad, Hercules, CA, USA), and normalized to GAPDH bands for heart and β-actin bands for the aorta.

### 4.10. Total NOS Activity

Total NOS activity was determined in crude homogenates of the heart and aorta by measuring the formation of [3H]-L-citrulline from [3H]-L-arginine (ARC, Saint Louise, MO, USA) [69]. Briefly, 50 µL of 20% homogenates were incubated in the presence of 0.5 M Tris-HCl, pH 7.4, 10 mM NADPH, 20 mM CaCl2, 100 µM [3H]-L-arginine), 1mg/mL calmodulin, 1:1 FAD/FMN, and 50 mM TH4 in a total volume of 100 µL. Incubation was carried out for 30 min at 37 °C. The reaction was then stopped by the addition of 1 mL of 0.02 M HEPES buffer pH 5.5, containing 2 mM EDTA, 2 mM EGTA, and 1 mM L-citrulline. The samples were applied to 1 mL Dowex 50WX-8 columns (Na + form). [3H]-L-citrulline was measured with a Quanta Smart TriCarb Liquid Scintillation Analyser (Packard Instrument Company, Meriden, CT, USA).

### 4.11. Statistical Analysis

Data are presented as mean ± SEM. One-way analysis of variance (ANOVA) and the Bonferroni test were used for statistical analysis. Values were considered significant with probability value *p* < 0.05 (both for ANOVA and Bonferroni test). *p* values were multiplicity adjusted.

## Figures and Tables

**Table 1:** Characteristics of pure and loaded nanoparticles (NP) determined with dynamic light scattering (DLS) of the NP.

	Diameter (nm)	PDI	Zeta Potentials (g)
NP	233 ± 14	0.03	−15.5
SIMV	347 ± 52	0.23	−29.2
CoQ10	270 ± 24	0.12	−15.8

SIMV, simvastatin; CoQ10, coenzyme Q10; PDI, size distribution.

**Table 2:** Body weight (BW), heart weight (HW), left kidney weight (LKW), relative heart weight as HW/tibia length (TL), and relative KW as KW/BW in the control group and groups treated with empty nanoparticles (NP), simvastatin-loaded nanoparticles (SIMV), a combination of simvastatin- and coenzyme-Q10-loaded nanoparticles (SIMV+CoQ10), and coenzyme-Q10-loaded nanoparticles (CoQ10).

	BW (g)	HW (g)	LKW (g)	Relative HW (HW/TL)	Relative KW (g/100 g BW)
Control	687.5 ± 18.5	1.3 ± 0.1×10^−2^	1.66 ± 10^−6^	0.032 ± 0.1 × 10^−2^	24 × 10^−6^ ± 10^−6^
NP	637.3 ± 17.2	1.2 ± 0.3 × 10^−3^	1.68 ± 2 × 10^−6^	0.031 ± 0.3 × 10^−3^	27 × 10^−6^ ± 2 × 10^−6^
SIMV	636.1 ± 26.9	1.2 ± 0.1 × 10^−2^	1.8 ± 2 × 10^−6^	0.031 ± 0.1 × 10^−2^	29 × 10^−6^ ± 2 × 10^−6^
SIMV+CoQ10	656.8 ± 8.3	1.3 ± 0.4 × 10^−2^	1.75 ± 3 × 10^−6^	0.033 ± 0.4 × 10^−2^	27 × 10^−6^ ± 3 × 10^−6^
CoQ10	639.8 ± 17.2	1.3 ± 0.1 × 10^−2^	1.67 ± 10^−6^	0.031 ± 0.1 × 10^−2^	26 × 10^−6^ ± 10^−6^

Data are means ± SEM from 6 animals in each group.

**Table 3:** Lipid profile and hepatic conjugated diene concentration in the control group and groups treated with empty nanoparticles (NP), simvastatin-loaded nanoparticles (SIMV), a combination of simvastatin-loaded and coenzyme-Q10-loaded nanoparticles (SIMV+CoQ10), and coenzyme-Q10-loaded nanoparticles (CoQ10).

	TG (mmol/L)	Total chol (mmol/L)	HDL (mg/dL)	LDL (mg/dL)	Hepatic CD (nmol/g Tissue)
Control	2.9 ± 0.2	7.7 ± 0.2	147.3 ± 10.1	70.9 ± 2.7	1424.1 ± 57.6
NP	5.5 ± 0.7 **	5.5 ± 0.4 *	96.1 ± 11.2 **	53.9 ± 6.6	1202.3 ± 32.1
SIMV	3.0 ± 0.3	4.9 ± 0.5 *	81.4 ± 7.6 ***	42.7 ± 4.6 *	1278.2 ± 31.7
SIMV+CoQ10	3.3 ± 0.5	6.0 ± 0.4	135.5 ± 8.7	45.0 ± 3.3 *	1132.3 ± 12.7 ***
CoQ10	2.9 ± 0.5	6.2 ± 0.5	143.7 ± 6.3	49.6 ± 4.1 *	1039.0 ± 46.0 ***

TG, triglyceride; total chol, total cholesterol; HDL, high-density lipoprotein; LDL, low-density lipoprotein; CD, conjugated dienes. * *p* < 0.05; ** *p* < 0.01; *** *p* < 0.001 compared to the control group. Data are means ± SEM from 6 animals in each group.

## Data Availability

Data supporting reported results can be found in archived datasets of the Department of Neuro-Cardiovascular Interactions, Institute of Normal and Pathological Physiology, Centre of Experimental Medicine, Slovak Academy of Sciences.
